# LncRNAs as regulators of chemoresistance and chemosensitivity in triple-negative breast cancer

**DOI:** 10.1590/1678-4685-GMB-2025-0242

**Published:** 2026-05-22

**Authors:** Miguel Ángel Cáceres-Durán, Anna Carolina Lima Rodrigues, Juscelino Carvalho de Azevedo, Caio Dantas Alves, Danielle Queiroz Calcagno, Bárbara do Nascimento Borges

**Affiliations:** 1 Universidade Federal do Pará, Instituto de Ciências Biológicas, Laboratório de Biologia Molecular, Belém, PA, Brazil.; 2 Instituto Agronômico de Campinas (IAC), Centro de Citricultura “Sylvio Moreira”, Cordeirópolis, SP, Brazil.; 3 Universidade Federal do Pará, Núcleo de Pesquisas em Oncologia, Belém, PA, Brazil.

**Keywords:** Breast cancer, therapeutic response, chemotherapy, precision medicine, ncRNA

## Abstract

Triple-negative breast cancer (TNBC) is the most aggressive molecular subtype of breast cancer, characterized by significant heterogeneity and high recurrence rates. Due to this heterogeneity, TNBC often develops resistance to chemotherapy, leading to aggressive clinical behavior and poor prognosis. Long non-coding RNAs (lncRNAs), defined as RNAs longer than 500 nucleotides that act at both transcriptional and post-transcriptional levels, influence therapeutic responses in TNBC through various molecular mechanisms, thereby directly impacting treatment effectiveness and patient outcomes. Importantly, lncRNAs have a dual, context-dependent role in TNBC, either promoting chemoresistance or enhancing chemosensitivity by functioning as competing endogenous RNAs, regulating epigenetic processes, stabilizing mRNAs and proteins, and affecting DNA damage repair, apoptosis, autophagy, epithelial-mesenchymal transition, and pro-survival signaling pathways. These functions indicate their potential as therapeutic targets and biomarkers for personalized treatment. In this review, we examine the biological functions of lncRNAs in TNBC, highlighting their molecular mechanisms and clinical significance. We also review evidence on how lncRNAs contribute to therapeutic resistance and sensitivity and explore emerging perspectives on their potential as biomarkers and therapeutic targets for TNBC management.

## Background

Breast cancer (BC) is the most common malignancy and the second leading cause of cancer-related death among women worldwide (Bray et al., 2024). Triple-negative breast cancer (TNBC), the most aggressive BC subtype, accounts for 15-20% of cases and occurs more frequently in premenopausal women, black women, and carriers of *BRCA1* germline mutations. TNBC is characterized by the absence of estrogen receptor (ER), progesterone receptor (PR), and human epidermal growth factor receptor 2 (HER2) expression, limiting treatment options mainly to chemotherapy and surgery (Allison et al., 2020; Bianchini et al., 2021). Clinically, TNBC is associated with a poor prognosis, high recurrence rates, and lower overall survival (Howlader et al., 2018; Darida et al., 2025).

Despite its aggressive clinical behavior, TNBC is a highly heterogeneous disease comprising distinct molecular subtypes that critically influence therapeutic response and clinical outcomes (Lehmann et al., 2016; Yin et al., 2020). Systemic treatment of TNBC is primarily based on cytotoxic chemotherapy, with anthracyclines inducing DNA damage through topoisomerase II inhibition, taxanes disrupting mitosis by stabilizing microtubules, and platinum-based agents promoting DNA crosslinking, particularly in tumors with impaired DNA repair capacity. In selected clinical settings, molecular profiling has enabled the use of targeted therapies, including PARP inhibitors for BRCA-mutated tumors and immune checkpoint inhibitors for PD-L1-positive disease, while antibody-drug conjugates and inhibitors of oncogenic signaling pathways are increasingly used in advanced or refractory TNBC (Bertucci et al., 2006; Bardia et al., 2017, 2019; Han et al., 2023; Savas et al., 2022; Riaz et al., 2025).

Although TNBC often shows a favorable initial response to systemic therapy compared with other BC subtypes, it frequently develops chemoresistance, which contributes to its aggressive behavior and presents a major therapeutic challenge (Bai et al., 2021). Chemoresistance is the ability of tumor cells to evade the cytotoxic effects of chemotherapy through phenotypic and molecular adaptations driven by various mechanisms, collectively leading to treatment failure and disease progression (Ji et al., 2019; Nath et al., 2021).

Mechanisms of chemoresistance in TNBC can be broadly categorized into three main categories. First, decreased intracellular drug accumulation often results from the overexpression of efflux transporters, such as P-gp (encoded by *MDR1*) and other ABC family members. Second, detoxification systems, such as enzymes like glutathione S-transferases (GSTs), can conjugate and deactivate cytotoxic agents. Third, improved DNA repair ability, through pathways like nucleotide excision repair (NER) and homologous recombination (HR), enables tumor cells to repair chemotherapy-induced DNA damage and stay alive (Ji et al., 2019; Lee et al., 2020; Singh and Reindl, 2021; Tian et al., 2023).

Besides these canonical processes, mutations in key signaling pathways, including PI3K/Akt/mTOR and RAS/MAPK/ERK, also confer proliferative and survival advantages, further reducing therapeutic effectiveness (Huang et al., 2011; Lønning and Knappskog, 2013; Antoun and Chioni, 2023). Overall, these changes highlight the complex, multifactorial nature of chemoresistance in TNBC and underscore the importance of molecular targeted treatment strategies.

Advancements in sequencing technologies have demonstrated that the dysregulation of non-coding RNAs (ncRNAs) plays a vital role in the development, chemoresistance, and progression of various cancers, including TNBC (Das et al., 2023). These ncRNAs are a heterogeneous group of RNA transcripts that are not translated into proteins but are involved in many physiological and pathological processes by regulating gene expression (Nemeth et al., 2023). Building on recent conceptual advances, Mattick et al. (2023) proposed a unified classification of ncRNAs into three main groups: (1) small RNAs (< 50 nt); (2) RNA polymerase III transcripts (~50-500 nt); (3) long ncRNAs (> 500 nt) (Mattick *et al.*, 2023). While short ncRNAs have been extensively studied in several diseases, long non-coding RNAs (lncRNAs) have recently emerged as key regulators, and their potential practical applications are increasingly being explored (Nemeth *et al.*, 2023).

Recently, it was shown that lncRNAs play a crucial role in TNBC development and in chemotherapy resistance or sensitivity, highlighting their potential as therapeutic targets and biomarkers for personalized treatment of the disease (Chai et al., 2023; Jiang et al., 2023). In this review, we provide an overview of the biological functions of lncRNAs in TNBC, emphasizing their underlying molecular mechanisms and clinical significance. We also examine their impact on therapeutic responses, especially their role in chemoresistance. Finally, we discuss emerging perspectives on the potential of lncRNAs as biomarkers and therapeutic targets for managing TNBC.

## Biological aspects and technical insights into long non-coding RNAs

LncRNAs constitute the largest and most functionally diverse group of ncRNAs. They are transcripts longer than 500 nucleotides that generally do not encode proteins but are associated with a wide range of biological functions. Unlike short ncRNAs, which mainly participate in gene silencing, lncRNAs exhibit several molecular mechanisms, contributing to their greater functional complexity. Based on their genomic context, lncRNAs are classified as sense, antisense, bidirectional, intronic, intergenic, and enhancer-associated lncRNAs (Valenti et al., 2017; Chi et al., 2019; Cáceres-Durán et al., 2020).

**Figure 1 -:**
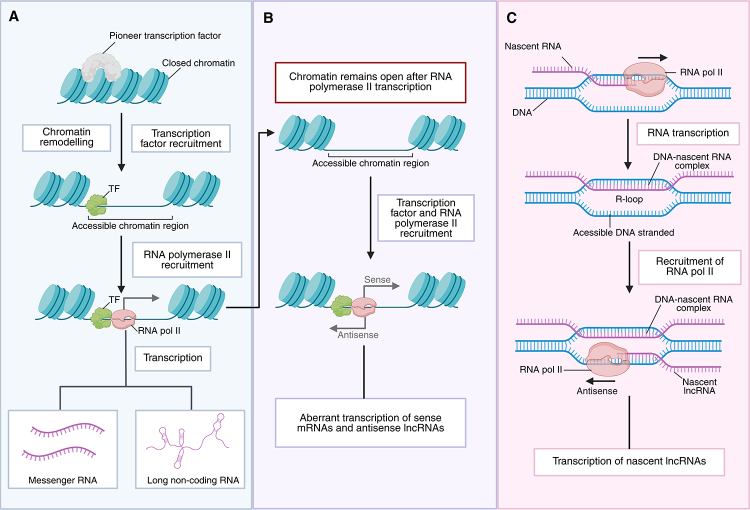
Mechanisms of lncRNA biogenesis. (A) Conventional transcription of coding and non-coding RNAs involves pioneer transcription factors binding to closed chromatin regions, promoting chromatin remodeling and recruiting RNA pol II. Transcription at accessible loci produces both mRNAs and lncRNAs. (B) In some cases, chromatin remains accessible after RNA pol II-mediated transcription, allowing transcription factors and RNA pol II to be re-recruited, resulting in the simultaneous synthesis of sense mRNAs and antisense lncRNAs from the same genomic region. (C) During transcription, the formation of a DNA-nascent RNA hybrid (R-loop) exposes single-stranded DNA, enabling additional recruitment of RNA pol II and the transcription of antisense or overlapping nascent lncRNAs. TF: Transcription factor; RNA pol II: RNA polymerase II; lncRNA: long non-coding RNA. Created in BioRender. Borges, B. (2026) https://BioRender.com/f09hr9c.

The biogenesis of lncRNAs shares considerable similarities with that of messenger RNAs (mRNAs), although they possess unique features that give them specific functions (Mattick et al., 2023). Usually, a pioneer transcription factor binds to closed chromatin, initiating its remodeling and the recruitment of RNA polymerase II, leading to the transcription of mRNAs and lncRNAs (Figure 1A). However, lncRNAs can also be transcribed through noncanonical mechanisms, such as re-recruitment of transcription factors and RNA polymerase II to genomic sites that remain transcriptionally permissive or dynamically accessible after transcription, or through R-loop-related processes (Figures 1 B-C) (Nojima and Proudfoot, 2022). Most lncRNAs are transcribed from DNA by RNA polymerase II, though some may also be produced by RNA polymerase I or III (Mattick *et al.*, 2023; Nojima and Proudfoot, 2022).

**Figure 2 -:**
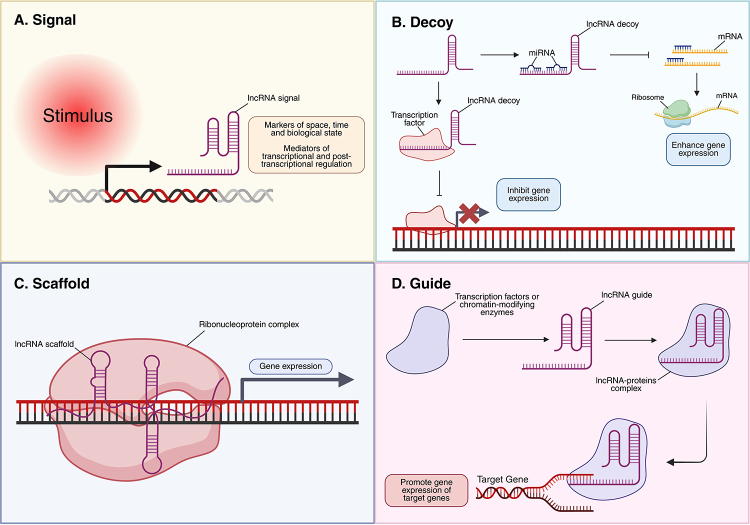
Classical molecular archetypes of lncRNAs. (A) Signal: lncRNAs are induced by specific stimuli, serving as markers of cellular condition, developmental stage, or environmental context, while also regulating gene expression at transcriptional and post-transcriptional levels. (B) Decoy: lncRNAs influence gene expression by sequestering transcription factors or sponging miRNAs, thus preventing repression or enhancing mRNA translation. (C) Scaffold: lncRNAs act as structural platforms for assembling ribonucleoprotein complexes that regulate gene expression. (D) Guide: lncRNAs attract transcription factors or chromatin modifiers, guiding them to specific genomic sites to control target gene expression. Created in BioRender. Borges, B. (2026) https://BioRender.com/mmmx4i8.

After transcription, the biological targets of lncRNAs are identified by specific signals, sequences, and structural domains within their primary, secondary, or tertiary conformations. This structural complexity allows lncRNAs to interact with DNA, RNA, and proteins, thereby modulating transcriptional and post-transcriptional processes through multiple mechanisms. Based on their diverse interactions and regulatory roles, lncRNAs are generally categorized into four molecular archetypes: signal, decoy, scaffold, and guide (Figure 2). They can act as signals of the cellular state (Figure 2 A ) or regulate gene expression through inhibition or activation (Figures 2 B-D ) (Wang and Chang, 2011; Liu et al., 2021; Chen and Kim, 2024).

Although the four classical molecular archetypes encompass a substantial proportion of reported lncRNA functions, the structural versatility of lncRNAs enables additional, context-dependent modes of action. Accordingly, several studies have described lncRNAs that enhance translation without altering mRNA abundance, mediate chromatin remodeling through covalent or non-covalent interactions, or contribute to protein stabilization by acting as molecular chaperones (Han and Chang, 2015; Liu et al., 2021; Wu et al., 2021; Takakuwa et al., 2023; Hu et al., 2024; Oo et al., 2025). These functions operate within, or in combination with, the classical archetypal framework rather than representing independent or exclusive archetypes.

High-throughput sequencing technologies have transformed transcriptome analysis by enabling the identification, annotation, and quantification of various RNA transcripts, including lncRNAs. RNA sequencing (RNA-seq) provides a comprehensive analysis of both coding and non-coding regions, which is especially useful for discovering new lncRNAs and alternative isoforms. On the other hand, microarray platforms with specific probes can be used for large-scale screening of already known lncRNAs (Shen et al., 2015; Liu et al., 2016; Chowdhary et al., 2021; Bagger et al., 2024). 

To validate findings from sequencing and array analyses, quantitative RT-PCR and Northern blotting - more specific, sensitive, and targeted methods - remain standard techniques for confirming gene expression levels independently (Shen et al., 2015; Liu et al., 2016; Tellez-Gabriel and Heymann, 2019; Jiang et al., 2023). In addition to validation, RNA fluorescence *in situ* hybridization (RNA-FISH) enables visualization of lncRNA distribution within cells and subcellular compartments. This is especially important because the subcellular localization of lncRNAs greatly influences their mechanisms of action: nuclear lncRNAs often regulate transcription and chromatin organization. In contrast, cytoplasmic lncRNAs generally impact mRNA stability or translation (Luo et al., 2024). Furthermore, new techniques leveraging RNase H activity or RNA-DNA hybrid recognition are being developed to detect and analyze individual lncRNAs in complex transcriptional environments (Bostick et al., 2016).

LncRNAs are commonly dysregulated in human cancers and contribute to tumor cell growth, proliferation, migration, invasion, metastasis, apoptosis, and angiogenesis. Increasing evidence indicates that lncRNAs also modulate cell sensitivity to chemotherapy, helping eliminate tumor cells by inhibiting cell growth and inducing apoptosis (Taghvimi et al., 2022). Considering their potential, targeting these molecules offers a promising approach to overcoming chemoresistance and restoring tumor drug sensitivity. 

## LncRNAs involved in resistance in TNBC

Substantial evidence indicates that lncRNAs are crucial regulators in the development and maintenance of resistance to various chemotherapeutic drugs (as summarized in Table 1). The increased expression of these lncRNAs is consistently associated with survival mechanisms that enable cancer cells to withstand therapeutic stress, operating through different molecular pathways. Among the most recent and significant mechanisms, we can highlight.

**Table 1 -:** LncRNAs associated with chemoresistance in Triple-Negative Breast Cancer (TNBC) and their main mechanisms of action. The “Expression” column indicates the expression pattern (↑ for upregulation) in resistant models. The “Target / Mechanism” column describes the pathway or molecule regulated by the lncRNA that contributes to the resistant phenotype.

Resistance Mechanism	LncRNA	Drug(s)	Expression	Target / Mechanism	Reference
CeRNA (“Molecular Sponge”)	*LINC00667*	Docetaxel	↑	Sponge for miR-200b-3p, preventing *BCL-2* inhibition and suppressing apoptosis.	Li et al., 2022
	*MCM3AP-AS1*	Doxorubicin, Docetaxel	↑	Sponge for miR-524-5p, leading to overexpression of the oncogene *RBM39*.	Wang et al., 2024
	*LINC00839*	Paclitaxel	↑	Sponge for miR-142-3p, resulting in overexpression of the epigenetic regulator BRD4.	Liang et al., 2025
	*OTUD6B-AS1*	Paclitaxel	↑	Sponge for miR-26a-5p, leading to MTDH overexpression and promoting genomic instability and autophagy.	Li et al., 2021
	*ZEB1-AS1*	Doxorubicin	↑	Sponge for miR-186-5p, increasing ABCC1/MRP1 expression and promoting drug efflux.	Lu et al., 2022
	*MALAT1*	Various	↑	Sponge for miR-140-5p, releasing JAG1 and VEGFA into the tumor microenvironment, promoting angiogenesis and resistance.	Liu et al., 2022
Epigenetic Modulation/ mRNA Stability	*LINC00115*	Paclitaxel	↑	Recruits SETDB1 to methylate and repress the tumor suppressor gene PLK3, stabilizing HIF-1α and activating genes such as *LDHA* and *MDR1*.	Luo et al., 2024
	*HIF1A-AS2/AK124454*	Paclitaxel	↑	Promote proliferation, invasion, and resistance, partly by attenuating drug-induced G2/M cell cycle arrest.	Jiang et al., 2016
	*DANCR*	Cisplatin, Paclitaxel	↑	Stabilizes transcription factor KLF5 via acetylation, repressing the tumor suppressor *P27* and promoting proliferation, invasion, and resistance.	Su et al., 2023; Nicolescu et al., 2024
	*DDIT4-AS1*	Paclitaxel	↑	Recruits AUF1 to stabilize DDIT4 mRNA, inhibiting the mTOR pathway, activating protective autophagy, and promoting cell survival.	Jiang et al., 2023
DNA Repair/ Evasion of Cell Death	*BORG*	Doxorubicin	↑	Activates the pro-survival NF-κB pathway and physically binds RPA1 to promote DNA repair after genotoxic stress.	Gooding et al., 2019b
	*LINP1*	Etoposide	↑	Stabilizes the DNA repair complex IGFBP-3/NONO/SFPQ, essential for efficient non-homologous end joining (NHEJ).	De Silva *et al.*, 2019
	*NEAT1*	Doxorubicin, Paclitaxel	↑	Reduces apoptosis, maintains cancer stem-like cells (CSCs), and may influence DNA repair pathways.	Shin et al., 2019
Epithelial-Mesenchymal Transition (EMT)	*DCST1-AS1*	Doxorubicin, Paclitaxel	↑	Promotes EMT by regulating ANXA1 and facilitating TGF-β/Smad pathway activation, enhancing invasion, migration, and metastasis.	Tang et al., 2020
	*Lnc005620*	Epirubicin	↑	Binds *ITGB1*, increasing integrin β1 levels and promoting cell adhesion and survival signaling.	Wang et al., 2021
	*AF178030.2*	Paclitaxel	↑	Binds and suppresses *TRPS1*, promoting EMT and therapeutic failure.	Zhao et al., 2022
Undefined mechanism	*SUMO1P3*	Various	↑	Found in serum exosomes and plasma; specific resistance mechanism not fully elucidated.	Na-Er et al., 2021
	*DRAIR*	Doxorubicin	↑	Detected in resistant cell lines, TNBC patient mammary tissue, and matched plasma. Molecular mechanism under investigation.	Wang et al., 2022

## Endogenous competitive RNA (ceRNA) mechanism

Among the most common mechanisms of chemoresistance, the role of endogenous competitive RNA (ceRNA) stands out, in which lncRNAs act as molecular sponges that sequester microRNAs (miRNAs), preventing them from silencing target mRNAs, often oncogenes or resistance genes. In docetaxel-resistant cells, *LINC00667* has been shown to act as a sponge for miR-200b-3p, thereby preventing the inhibition of the anti-apoptotic gene *BCL-2*. The resulting increase in Bcl-2 protein suppresses apoptosis and confers chemoresistance. In TNBC cell lines such as MDA-MB-231 and MDA-MB-468, *LINC00667* was significantly elevated, especially in exosomes derived from resistant cells, which could transfer the resistant phenotype to sensitive cells. Functionally, silencing this lncRNA restored chemosensitivity, reduced cell viability, and increased cleaved caspase-3 and caspase-9 levels (Li et al., 2022). Another lncRNA, *MCM3AP-AS1*, also promotes docetaxel resistance, in addition to doxorubicin, by sponging miR-524-5p and thereby upregulating the oncogene *RBM39* (Wang et al., 2024).

LncRNAs are also involved in paclitaxel resistance. *LINC00839* has been shown to act as a sponge for miR-142-3p, resulting in the overexpression of the epigenetic regulator *BRD4* and contributing to drug resistance (Liang et al., 2025). Similarly, *OTUD6B-AS1* sequesters miR-26a-5p, leading to *MTDH* overexpression, disruption of autophagy, and increased genomic instability-mechanisms that together enhance paclitaxel resistance (Li *et al.*, 2021). In the context of doxorubicin resistance, *ZEB1-AS1* has been identified as a ceRNA for miR-186-5p, thereby increasing ABCC1/MRP1 expression and promoting drug efflux (Lu et al., 2022). Notably, *MCM3AP-AS1* overlaps with this mechanism, contributing to resistance to doxorubicin and docetaxel (Wang et al., 2024).

Other lncRNAs can spread resistance traits more widely. *MALAT1*, for example, when secreted in exosomes, acts as a sponge for miR-140-5p, thereby releasing JAG1 and VEGFA into the tumor microenvironment, promoting angiogenesis and aiding resistance mechanisms (Liu et al., 2022).

## Epigenetic modulation and mRNA stability

In addition to miRNA sequestration, lncRNAs can directly regulate gene expression through epigenetic modulation or by stabilizing mRNAs and proteins. The lncRNA *LINC00115* is upregulated by paclitaxel. Its molecular mechanism involves recruiting the histone methyltransferase SETDB1 to methylate and epigenetically repress the *PLK3* gene, a tumor suppressor kinase. The resulting reduction in PLK3 activity stabilizes the hypoxia-inducible factor alpha subunit (HIF1α), leading to the transcription of pro-tumorigenic target genes, such as *LDHA* (involved in glycolytic metabolism) and *MDR1*, thereby promoting resistance and metastasis (Luo et al., 2024). Furthermore, another study identified a transcriptomic signature comprising the lncRNAs *HIF1A-AS2* and *AK124454*, both overexpressed in TNBC and linked to shorter recurrence-free survival. This signature stratified patients into high- and low-risk groups, outperforming traditional clinical parameters. Functionally, *HIF1A-AS2* and *AK124454* were shown to promote proliferation, invasion, and paclitaxel resistance, partly by attenuating drug-induced G2/M phase cell arrest, positioning them as predictive biomarkers of taxane resistance and potential therapeutic targets (Jiang et al., 2016). In post-transcriptional regulation, *DANCR* confers resistance to cisplatin and paclitaxel in cell lines such as BT549 and MDA-MB-231 by stabilizing the transcription factor KLF5 through stimulating its acetylation, which represses the tumor suppressor gene *P27*, leading to increased cell proliferation, invasion, and drug resistance (Su et al., 2023; Nicolescu et al., 2024).

Modulation of autophagy and transcription factors constitutes another key axis. Investigations using reliable models, including TNBC cell lines (BT549, HCC1806, MDA-MB-231, MDA-MB-436), luminal models (MCF-7, T47D), and normal mammary epithelial cells (MCF10A) for comparison, as well as mice and patient-derived organoids, have shown that *DDIT4-AS1* causes paclitaxel resistance. Its mechanism involves recruiting the RNA-binding protein AUF1 (hnRNPD), which stabilizes *DDIT4* mRNA. The DDIT4 protein inhibits the mTOR pathway, thereby triggering protective autophagic flux and supporting homeostasis and survival of cancer cells under treatment stress (Jiang et al., 2023).

## DNA repair and evasion of cell death

The ability to repair chemotherapy-induced DNA damage is a key mechanism of resistance, and several lncRNAs are closely involved in this process. Studies using *in vitro* models (4T1 and D2.OR cell lines) and *in vivo* (mice) have identified the lncRNA *BORG* (*BMP/OP*-Responsive Gene) as a potent mediator of doxorubicin resistance. *BORG* promotes activation of the pro-survival NF-κB signaling pathway and directly interacts with RPA1, an essential component of the DNA repair process, helping cells recover after genotoxic stress (Gooding et al., 2019 a ). Interestingly, pharmacological treatments, such as bortezomib (0.5 nM) in D2.OR cell lines with high *BORG* levels restored sensitivity to doxorubicin, indicating potential therapeutic options (Gooding *et al.*, 2019a, b).

Additionally, in the HCC1806 BC cell line, the lncRNA *LINP1* is overexpressed in response to etoposide resistance, a topoisomerase inhibitor. *LINP1* stabilizes a key protein complex composed of IGFBP-3, NONO, and SFPQ, which is crucial for the effectiveness of non-homologous end-joining (NHEJ)-mediated DNA repair, thereby leading to resistance (De Silva *et al.*, 2019). Furthermore, *NEAT1* is linked to resistance to doxorubicin and paclitaxel, not only by reducing apoptosis but also by maintaining cancer stem cells and possibly influencing DNA repair pathways (Shin et al., 2019).

## Epithelial-mesenchymal transition (EMT)

Epithelial-mesenchymal transition (EMT), a process closely linked to the acquisition of resistance and metastatic potential, is also regulated by lncRNAs. *DCST1-AS1,* which is overexpressed in doxorubicin- and paclitaxel-resistant cells (MDA-MB-231, BT-549, T-47D, and MCF7), actively promotes EMT. It facilitates the activation of the TGF-β/Smad signaling pathway by regulating ANXA1 (Annexin A1), thereby increasing invasion, migration, and metastasis (Tang et al., 2020). 

Similarly, in the MDA-MB-231 cell line, *LNC005620* promotes epirubicin resistance by binding to and increasing *ITGB1* expression, which encodes integrin β1. Elevated levels of β1 integrin enhance cell adhesion and survival signaling, reducing chemotherapy-induced apoptosis (Wang et al., 2021). Additionally, QSOX2, an overexpressed protein in TNBC involved in the EMT pathway and linked to poor prognosis, stabilizes ITGB1 (Kim et al., 2024).

Conversely, knocking down *AF178030.2*, an overexpressed lncRNA in TNBC paclitaxel-resistant cell lines MDA-MB-231 and MDA-MB-436, increases their sensitivity to this chemotherapeutic drug (Zhao et al., 2022). This lncRNA can directly bind to and suppress *TRPS1*, thereby promoting EMT by disrupting the regulation of several genes in this pathway (Rangel et al., 2016).

## LncRNAs involved in drug sensitivity in TNBC

In contrast to chemoresistance, chemosensitivity refers to the susceptibility of tumor cells to the cytotoxic effects of chemotherapeutic agents, a fundamental condition for treatment success. Complete response, or remission, is defined by the absence of detectable cancer and the normalization of disease markers. Although targeted molecular therapy shows significantly improved clinical efficacy and safety, complete responses in advanced-stage cancers are rare (Tyner et al., 2022). In this context, lncRNAs emerge as key regulators of chemosensitivity in TNBC, acting through epigenetic and post-transcriptional mechanisms.

The lncRNA *SNHG10* exhibits a sensitizing effect by promoting demethylation of the miR-302b promoter, thereby increasing apoptosis and decreasing cell proliferation in response to doxorubicin (Aini et al., 2022). Similarly, *LINC-PINT* functions as a tumor suppressor by aiding the breakdown of the pro-resistance protein NONO, thereby restoring paclitaxel sensitivity (Chen et al., 2020). *CARMN*, another lncRNA with lower expression in TNBC, contains miR-143-3p, which inhibits MCM5 expression-a crucial regulator of DNA replication-enhancing the cytotoxic effects of cisplatin (Sheng et al., 2021). These mechanisms emphasize the therapeutic potential of lncRNA modulation in reversing resistant phenotypes. 

The precise regulation of chemosensitivity also involves lncRNAs that modulate programmed cell death pathways. *MEG3* is a key mediator of cisplatin-induced pyroptosis by activating the NLRP3/caspase-1/GSDMD pathway, and its overexpression predicts a positive therapeutic response (Yan et al., 2021). On the other hand, *MALAT1* has emerged as a significant factor that impairs chemosensitivity, promoting various resistance mechanisms by activating cell-survival pathways and “sponging” suppressive miRNAs. Evidence indicates that silencing *MALAT1*-either through genetic methods such as CRISPR/Cas9 or natural compounds such as MQG-significantly increases intracellular drug accumulation, triggers apoptosis, and reverses resistance (Shaath et al., 2021; Abdel-Latif et al., 2022; Adewunmi et al., 2023). Notably, deleting *MALAT1* also affects networks of other lncRNAs (*NEAT1, USP3-AS1, LINC-PINT),* implying a complex, interdependent regulatory system that governs therapeutic sensitivity (Shaath *et al.*, 2021).

The development of lncRNA-based signatures has led to significant progress in predicting therapeutic responses and personalized treatment approaches. The signature composed of *BPESC1*, *WDR72*, and *GADD45A* shows high sensitivity and specificity in predicting a complete pathological response to neoadjuvant chemotherapy in TNBC (Wang et al., 2018). Similarly, signatures derived from lncRNAs related to necroptosis (*ALMS1-IT1*, *LINC00485*, *HCP5*) or cellular stemness (*AC245100.6*, *LINC02511*, *FRGCA*) are associated with response patterns to specific chemotherapies and the tumor immune microenvironment (Xie et al., 2022; Zhang et al., 2024). Innovative therapeutic strategies have leveraged this knowledge, such as nanocomplexes for co-delivering antisense oligonucleotides targeting oncogenic lncRNAs (*ASBEL*) and chemotherapeutic agents (curcumin), demonstrating improved efficacy in preclinical models (He et al., 2023 a ). Likewise, pharmacological modulation of lncRNA-miRNA axes-such as the upregulation of *LOC554202*/miR-31 by potassium piperonate-shows synergistic effects with conventional chemotherapies, offering promising strategies to overcome resistance (Wang *et al.*, 2018; Tian et al., 2022). These advances underscore the translational potential of lncRNAs not only as biomarkers but also as therapeutic targets to enhance chemosensitivity in TNBC.

## Conclusions and future perspectives 

Growing evidence on the functions and expression levels of lncRNAs in TNBC offers new insights into the molecular mechanisms behind chemoresistance and chemosensitivity, guiding the development of personalized treatment strategies for TNBC patients. While the ability of lncRNAs to act as sponges for specific miRNAs and to modify metabolic pathways has been studied in TNBC tissues and *in vitro* models, the main challenge remains fully understanding the molecular mechanisms that regulate their functions and interactions with other ncRNAs (miRNAs and circRNAs) and proteins, which remain largely unclear. Similarly, the interactions of lncRNAs with the immune system, tumor microenvironment, microbiome, hormonal environment, and metabolome are still poorly understood.

Chemoresistance in TNBC remains a significant challenge for effective treatment, particularly in patients with metastatic disease. In this review, we highlighted that specific lncRNAs influence therapeutic resistance through various mechanisms, and that silencing them can reverse multidrug resistance by promoting programmed cell death. Additionally, several lncRNAs have been linked to chemosensitivity in TNBC, emphasizing their role in controlling drug response. Finally, the molecular signature of specific lncRNAs, such as *HIF1A-AS2* and *AK124454*, whether alone or combined with mRNA expression signatures, can be used to predict pathological complete response to neoadjuvant chemotherapy (Jiang et al., 2016; He et al., 2024).

**Figure 3 -:**
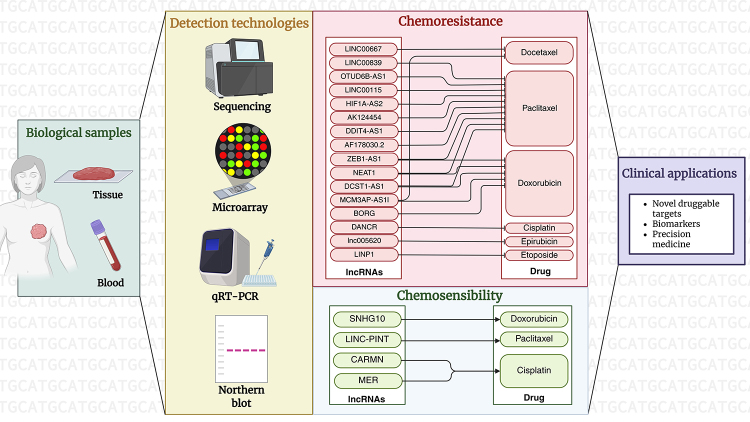
Clinical applications of lncRNAs in triple-negative breast cancer (TNBC). Tissue or blood samples can be used to analyze lncRNA expression using various detection methods, including sequencing, microarray, qRT-PCR, and Northern blot. These methods help identify predictive biomarkers linked to chemoresistance and chemosensitivity to different drugs. Such findings may lead to the discovery of new drug targets and biomarkers, as well as to advancements in precision medicine. Created in BioRender. Borges, B. (2026) https://BioRender.com/zw8lert.

Additionally, TNBC tumors exhibit high heterogeneity and clonal evolution, which cannot be effectively captured by traditional tissue biopsy, which provides only a single snapshot of the disease’s molecular status. Therefore, liquid biopsy has emerged as a minimally invasive method for detecting tumor-derived molecules in various body fluids, especially blood, and is used in the clinical management of TNBC patients (de Azevedo Jr et al., 2025). This approach shows promise for detecting circulating lncRNAs in these patients. Multiple studies have shown that lncRNAs can be found in serum and/or plasma and may serve as biomarkers for diagnosing and predicting outcomes in TNBC, including *BRE-AS1* (Gao et al., 2021), T376626 (He et al., 2023b), *TINCR* (Wang et al., 2020), and *XIST* (Lan et al., 2021). Some lncRNAs, such as *LINC00989* (Zhu et al., 2023), are used solely for prognosis. Likewise, chemoresistance might be linked to high levels of *SUMO1P3* (found in serum exosomes and plasma) (Na-Er et al., 2021) and *DRAIR* (present in doxorubicin-resistant cell lines, mammary tissue from TNBC patients, and matched plasma) (Wang *et al.*, 2022) (Figure 3).

Taken together, these findings indicate that lncRNAs are not only crucial predictive biomarkers of chemoresistance but also promising therapeutic targets to enhance chemosensitivity in TNBC. Approaches that silence oncogenic lncRNAs or mimic tumor suppressor lncRNAs represent an innovative frontier for developing combination therapies to overcome resistance and improve clinical outcomes in TNBC. Ultimately, the studies presented here offer valuable insights for future cancer research and clinical applications, aiming to improve therapeutic management for patients with TNBC.

## Data Availability

This is a review article and does not include original data generated or analyzed by the authors. All information discussed is based on previously published literature cited throughout the text.
